# Prevalence of obesity in panama: some risk factors and associated diseases

**DOI:** 10.1186/s12889-015-2397-7

**Published:** 2015-10-21

**Authors:** Anselmo Mc Donald, Ryan A. Bradshaw, Flavia Fontes, Enrique A. Mendoza, Jorge A. Motta, Alberto Cumbrera, Clara Cruz

**Affiliations:** Department of Research in Health Systems, Environment and Society, Gorgas Memorial Institute for Health Studies, Justo Arosemena Avenue and 35th Street, Panama, Panama; Department of Nutrition, Ministry of Health of Panama, Gorgas Avenue, Ancon, Panama; Gorgas Memorial Institute for Health Studies, Panama, Panama; School of Statistics, Faculty of Sciences, University of Panama, Avenida Transístmica, Panama, Panama

**Keywords:** Prevalence of obesity in Panama, Physical inactivity, Family history of obesity, Consumption of beverages / foods rich in sugar, Obesity-related diseases

## Abstract

**Background:**

To estimate the prevalence of obesity in Panama and determine some risk factors and associated diseases in adults aged 18 years and older.

**Methods:**

A cross-sectional descriptive study was conducted in the provinces of Panama and Colon where 60.4 % of all Panamanians 18 years or older reside, by administering a survey regarding the consumption of protective and predisposing foods and assessing the development of obesity by measuring the weight, height, and waist circumference of 3590 people. A single-stage, probabilistic, and randomized sampling strategy employing multivariate stratification was used. Individuals with a body mass index ≥ 30 kg/m^2^ (men and women) were considered obese. Prevalence and descriptive analysis were conducted according to sex using Odds Ratio, with statistical significance set at a p value ≤ 0.05.

**Results:**

The general prevalence of obesity was 27.1 % (30.9 % women and 18.3 % men). In women, obesity was associated with living in urban areas, being 40–59 years of age, being Afro-Panamanian, consuming beverages / foods rich in sugar, being physically inactive and having a family history of obesity. In men, obesity was associated with living in urban areas, consuming beverages/foods rich in sugar, and having a family history of obesity. Almost the totality of obese women (97.9 %), and 80.0 % of men with obesity had abdominal obesity according to the WHO classification. In both sexes, obesity was a risk factor associated to type 2 Diabetes Mellitus, hypertension, LDL values ≥ 100 mg/dL, and low HDL values (<50 mg/dL for women and < 40 mg/dL for men), Odds Ratio > 1.0; *P* < 0.05.

**Conclusions:**

Obesity represents a very serious threat to Panamanian public health. Our study confirms a direct association in Panama between excess weight, hypertension, type 2 Diabetes Mellitus, LDL values ≥ 100 mg/dL and low HDL values for women and men (<50 mg/dL and < 40 mg/dL, respectively). Intervention / treatment programs should be targeted, specially, to Afro-Panamanian women, whom are 40–59 years old, living in urban areas, and those having a family history of obesity.

## Background

Obesity is a global public health problem that, according to global indicators, has been increasing in both industrialized and underdeveloped countries [1–3] to the extent that the World Health Organization (WHO) identified it as a worldwide epidemic [4]. The WHO defines obesity as a Body Mass Index (BMI) greater than or equal to 30 kg/m^2^ [1, 5, 6].

Overweight/obesity is one of the highest causes of premature and avoidable deaths, second only to tobacco [5, 7, 8]. Globally, 2.8,000 000 individuals die each year as a result of complications of being overweight or obese. Mortality rates increase with increasing degrees of overweightness [3]. In the last 10 years, diseases related to obesity, remain in the top ten causes of avoidable deaths in Panama [9].

Prior to our investigation, there was no statistically representative data, of population researches that would prove the magnitude of the problem that obesity represents to the Panamanian public health system. Moreover, there is no available data, in Panama, about obesity’s associated risk factors, nor its associated diseases [1–4, 6, 8, 10–12].

Indeed, diseases related to obesity (cardiovascular diseases, Type 2 Diabetes Mellitus (T2DM), hypertension, and cancer) represented 49.4 % (8 782/17 767) of the total number of registered deaths on 2 013 [13].

Thus, the objective of this research was two-fold: 1) to estimate the prevalence of obesity in Panama and 2) determine some risk factors and associated diseases in Panamanian adults aged 18 years and older.

## Methods

### Study design and area

The data used for this article was obtained from database of the first Panamanian Survey on Risk Factors Associated with Cardiovascular Disease (PREFREC, by its acronym in Spanish). It was a cross-sectional, descriptive study conducted in the trans-isthmian zone of the Republic of Panama, between October 2 010 and January 2 011, developed by researchers from the Gorgas Memorial Institute (GMI) and from the Panamanian Ministry of Health.

**Fig. 1:**
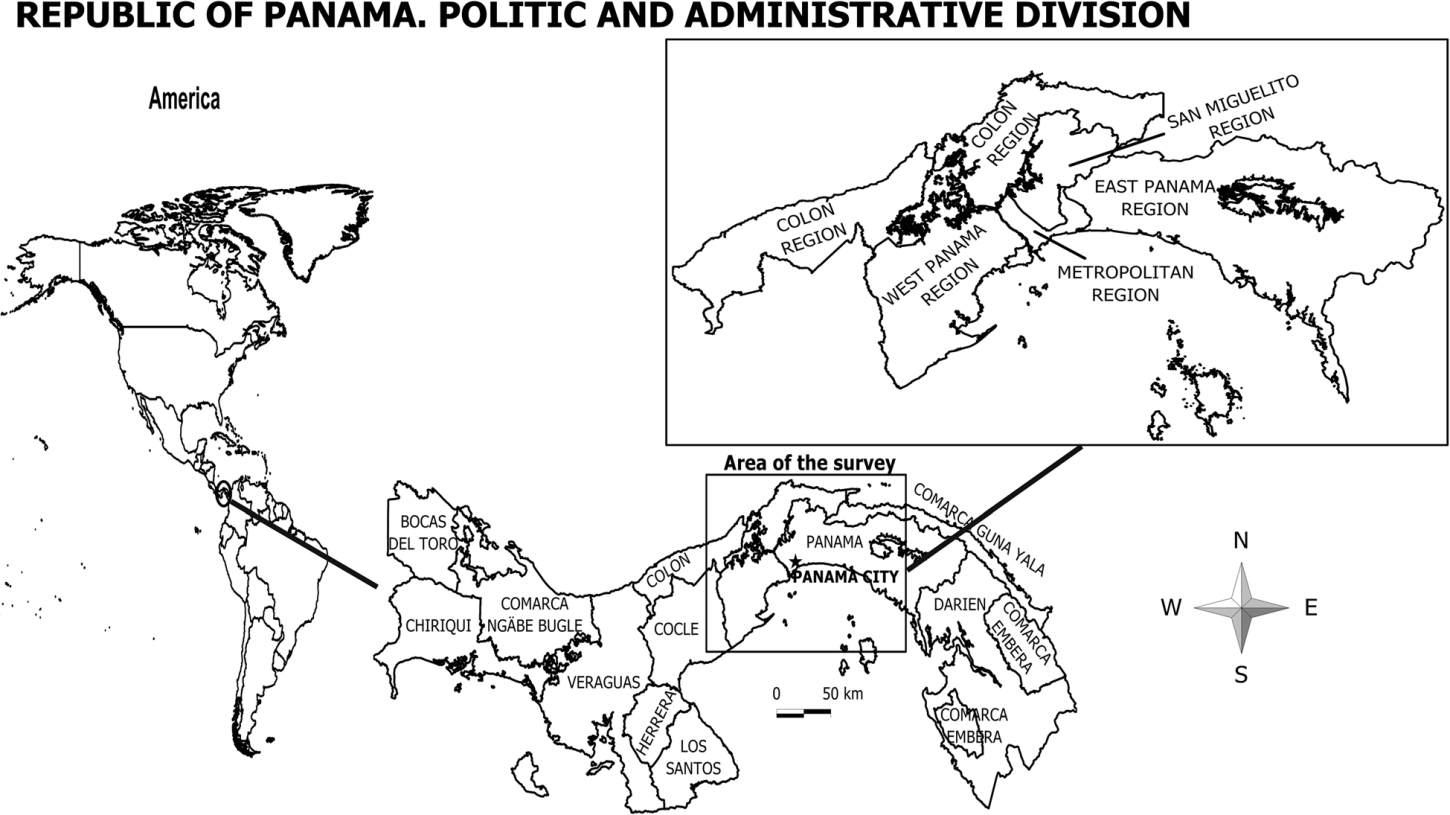
Republic of Panama. Political and administrative divisions

The study area encompassed the provinces of Panama and Colon, which contains the capital of Panama, and the health regions of Western Panama, San Miguelito, East Panama, Colon, and Metropolitan health region (Fig. 1) [14]. This provinces lodge the highest population in the country, where 60.4 % of all Panamanians 18 years or older reside. According to the National Institute of Statistics and Census (INEC, by its acronym in Spanish) the distribution of age, sex and other socio-demographic factors are similar in the remaining 39.6 % of the Panamanian population, not addressed in this research.

### Study sample population

The study included individuals aged 18 years and older who resided in private houses (according to maps produced by the national census for the year 2 000). Houses were sampled, employing a single-stage, probabilistic, and randomized sampling strategy with a multivariate stratification, developed by the INEC. This strategy was conducted separately for urban, rural and indigenous areas [14, 15]. Each census unit comprised private occupied houses (8–30 houses were reached per unit), which were stratified, on the first hand, by the Administrative Political Code of the Republic of Panama. Subsequently, they were stratified according to population size and the educational level of the study population [14, 16].

This survey was conducted applying a structured form (questionnaire), that consisted of 14 parts: parts 1 and 2 were disposed for socio-demographic variables; parts 3 through 6 were destined to learn the behavior of the participants towards the consumption of tobacco, intake of alcohol, consumption of foods and performance of physical activity, respectively; parts 7 through 11 were destined to learn about the personal pathologic history of hypertension, T2DM, high cholesterol, obesity, and stroke; part 12 was disposed to learn the participant’s family history of non-communicated chronic diseases; and part 13 was destined for the anthropometric measurements and for the results of fasting blood samples.

Professionals and students in their senior year of Health Sciences education (medical doctors, nurses, and nutritionists) administered the questionnaire. These collaborators were trained by the authors of the research in interviewing and survey management in order to guarantee the quality of the data collection process. Nutritionists (licensed and students) that collaborated in the survey were trained in anthropometric measurements. In indigenous areas, were dialects are spoken, the survey administrators were supported by interpreters who spoke these indigenous dialects [16].

It is important to stand out that nutritionists (licensed and students) were in charge of administrating parts 4 and 13 of the survey; the other parts were administered my medical doctors, and nurses (licensed professionals and students).

Fifteen days before the survey started, the population segments were visited (pre-screen procedure), in order to guarantee an adequate response rate and that the participants would be fasting. Using a spiral technique, a random survey was conducted between people aged 18 year and older residing in occupied houses that agreed to participate in the research (maximum 2 adults per household).

Potential participants were provided with material explaining the objective of the study, confidentiality of their participation, voluntary nature of the research, potential risks and benefits from their participation, fasting requirements, institutions to call to solve their doubts and were provided with a written form (Informed Consent) for them to sign in case they agree to participate in the study.

A total of 3 590 individuals aged 18 years and older who agreed to participate in the study were evaluated from the sampled houses. Pregnant women, individuals without weight and height values, and without waist circumference values, were excluded from this analysis, resulting in a sample size of 3 507.

### Variables

Obesity is our main variable, and was defined as all individuals with BMI ≥ 30 kg/m^2^ (men and women) [5, 7, 8]. For the purpose of calculating the BMI, height and weight measurements were done twice. The height was taken setting the participant in the Frankfurt plane; the participant was weighted being in light clothes, bare-foot, with both arms resting at each side of the body, and in the Frankfurt plane. If the difference between the two measurements was greater than 0.5 cm or 0.5 kg, a third measurement was taken. SECA® height measuring instruments and weighing scales were used.

Age was defined as the years from the time of the individual’s birth until the day the survey was conducted; sex, by the phenotypical characteristics that distinguish men from women; area, by the geographic domain where the respondent usually lives (urban, rural or indigenous); and ethnic group, by the participant’s self identification as an afro-Panamanian (Panamanian of African descent), mestizo, white, native American (Amerindian), or of Asian descent.

Physical activity was defined as any activity that requires energy expenditure under aerobic respiration, like sports and physical exercises and was classified into two categories: insufficient physical activity (less than 150 min/week) and physically active (150 min and more/week).

To identify the intake of predisposing foods to the development of obesity, a questionnaire of food consumption was developed and validated by licensed dietitians. In this questionnaire a variety of foods were listed, allowing the interviewee to indicate their daily frequency of food intake during a regular week. According to the frequency of weekly consumption of food reported by the interviewed individuals, the average daily intake was estimated.

The total of listed foods were classified into 4 groups, two “cardiovascular protective food” groups (a group of consumption of vegetables and fruits, and a group of consumption of non-fried fish and tuna fish) and two “cardiovascular risk food” groups (a group of consumption of sugary foods and a group of consumption of fried foods).

The method used to measure the frequency of consumption of protective foods and risk foods allowed us to identify the types of foods for which caloric density is associated with obesity and it permitted us to quantify the number of times they were consumed and by whom [15]. On this matter, excessive consumption of high fat foods was defined as the intake of 2 or more daily fried foods or foods high in fat, such as: “tortilla”, puff pastry, patty, chips, fried plantain, fried yucca, fried sausage, fried nuggets, fried chicken, fried meat, fried fish, fried pork and pork crackling; snacks, as “kaprichitos”, “doritos” and “taquitos”; and use of coconut milk or coconut oil for cooking.

Intake of foods or beverages with an equivalent of 6 teaspoons of sugar per day or more, such as sodas, artificial drinks (like Tang, Kool-Aid, etc.), milk chocolate bars, sweets, and candies in quantities of 6 or more, were considered as excessive consumption of beverages or foods high in sugar.

The biological variables included for this analysis were: the family history of obesity, abdominal obesity, T2DM, Hypertension (high blood pressure), Low Density Lipoprotein Cholesterol (LDL), and High Density Lipoprotein Cholesterol (HDL).

Family history of obesity was defined as the background of obesity in parents, siblings, aunts, uncles or grandparents in first or second degree of relatedness.

Waist circumference was measured placing the measuring tape around the patient’s abdomen (the patient should be standing with his arms separated from his core) between the uppermost border of the iliac crest and the lower border of the costal margin. The participant was requested to make a deep inspiration followed by a normal expiration; at this moment the tape was placed in the position discussed above and locked. The reading was taken twice. If the difference between these two was more than 1 cm, a third measurement was performed. SECA® measuring tape was used. In this sense, abdominal obesity was defined as a waist circumference > 88 cm in women and > 102 cm in men. Additionally, for men, we used the waist circumference cutoff point > 94 cm [16].

T2DM, was defined as those individuals who reported to be under pharmacological treatment for T2DM, and those who weren’t under pharmacological treatment but who presented fasting blood glucose values ≥ 126 mg/dL and/or glycated hemoglobin percentage (HbA1c) ≥ 6.5 % [14].

The measurement of blood pressure (BP) was performed with calibrated automatic sphygmomanometers made by American Diagnostic Corporation Model 6013, which had cuff sizes for people with normal weight and for people who were obese. Three BP measurements were made in the right arm of each person with the participant seated and with the minimum of 5 minutes between the start of the survey and the first measurement, and between the 2^nd^ and 3^rd^ measurements. An average of these 3 BP’s was used to define the participant’s BP [16]. On this matter, hypertension, involved those individuals who reported to be under pharmacological treatment for hypertension, and those who weren’t under pharmacological treatment, but who presented systolic blood tension values ≥ 140 mmHg and/or diastolic blood tension values ≥ 90 mmHg [16].

LDL and HDL values were obtained through analysis of blood samples, which were draw after the survey was applied. Persons with high levels of LDL included individuals with LDL values ≥ 100 mg/dL. Persons with low levels of HDL included women with HDL values of < 50 mg/dL and men with HDL values of < 40 mg/dL [17].

### Analysis plan

The general and specific prevalence in this study were estimated using percentages with 95 % confidence intervals (CI) and comparisons were made with the age-adjusted rates for the Panamanian population in 2014 [9]. Standard deviation (SD) was used to report averages.

For risk analysis Odds Ratio (OR) and P values were calculated using contingency tables for all variables. A P value < 0.05 was considered statistically significant. Multivariate logistic regression analysis was applied to the data set of the explanatory variables of obesity in men and women only to identify statistically significant variables [18, 19].

The data were processed using SPSS (version 19) (SPSS Inc, Chicago, IL), Microsoft Excel 2010 (Microsoft Corporation, Washington DC), and Epi Info, version 7.0 (Centers for Diseases Control and Prevention, Atlanta, GA).

All of the participants signed an informed consent form. The National Bioethics Committee of the Republic of Panama approved the research.

## Results

A total of 3 507 adults aged 18 years and over were studied, of which 1 057 (30.1 %) were male and 2 450 (69.9 %) were female. The average age was 45 ± 16 SD (45 ± 17.1 SD for men and 45 ± 15.9 SD for women) and median age was 45 years old (both for men and women).

### Obesity in whole sample

The prevalence of obesity was 27.1 % (CI 25.5–28.5) and was significantly associated with the following socio-demographic and biological variables: being female, people living in urban areas, among individuals between 30 and 59 years old, in Afro-Panamanians, in individuals who consumed beverages/foods rich in sugars (equivalent to 6 or more teaspoons of sugar per day), in those who engaged less than 60 min of physical activity per week, and in those with a family history of obesity, *P* < 0.05 - Table 1.

**Table 1:** Prevalence of obesity and associated factors in Panama

Variables	Prevalence of obesity (%)	Confidence Intervals (95 %)	Age-adjusted prevalence rates^a^	O.R.^b^	*P* value
TOTAL	27.1 (950 / 3507)	25.5–28.5	26.3	NA	NA
Sex					
Women	30.9 (757 / 2450)	29.2–32.8	29.8	2.00 (1.68–2.39)	*P* < 0.0001
Men	18.3 (193 / 1057)	15.7–20.3	18.4	0.50 (0.42–0.60)	*P* < 0.0001
Area					
Urban	31.5 (521 / 1655)	28.8–33.2	30.1	1.52 (1.31–1.77)	*P* < 0.0001
Rural	24.1 (399 / 1655)	21.9–26.1	24.0	0.75 (0.64–0.87)	*P* < 0.001
Indigenous	15.2 (30 / 197)	10.2–20.2	14.6	0.47 (0.31–0.70)	*P* < 0.001
Age (years)					
18–29	17.7 (124 / 701)	15.2–20.8	17.7	0.52 (0.42–0.64)	*P* < 0.001
30–39	30.4 (207 / 675)	26.5–33.5	30.7	12.1 (10.6–13.8)	*P* < 0.0001
40–49	34.2 (242 / 708)	30.5–37.5	34.2	1.53 (1.28–1.84)	*P* < 0.0001
50–59	30.7 (200 / 652)	27.5–34.5	30.7	1.24 (1.03–1.50)	*P* < 0.05
60 and over	23.0 (177 / 771)	20.0–26.0	23.0	0.76 (0.62–0.92)	*P* < 0.05
Ethnic groups					
Afro-Panamanian	36.7 (273 / 744)	33.5–40.5	35.9	1.79 (1.50–2.12)	*P* < 0.0001
Mestizo	26.7 (505 / 1894)	25.0–29.0	25.9	0.95 (0.82–1.11)	*P* = 0.50
Native	14.3 (54 / 378)	10.5–17.5	14.5	0.42 (0.30–0.57)	*P* < 0.0001
White	25.1 (105 / 418)	20.8–29.2	25.3	0.89 (0.70–1.13)	*P* = 0.33
Asian/Others/Not specified	17.8 (13 / 73)	9.0–26.6	18.6	0.58 (0.30–1.09)	*P* = 0.09
Intake of fatty foods					
2 or more fried food daily	27.3 (881 / 3227)	25.5–28.5	26.6	1.13 (0.85–1.51)	*P* = 0.39
Less than 2 fried food daily	24.9 (70 / 281)	19.7–29.7	22.0	0.88 (0.67–1.17)	*P* = 0.39
Intake of beverage / foods rich in sugars					
6 teaspoons per day or more	30.1 (396 / 1314)	27.5–32.5	30.0	1.28 (1.09–1.49)	*P* < 0.05
Less of 6 teaspoons per day	25.3 (554 / 2193)	23.4–29.6	23.8	0.78 (0.67–0.91)	*P* < 0.05
Physical Activity					
Physically active	19.9 (85 / 426)	16.2–23.8	20.1	0.64 (0.49–0.83)	*P* < 0.001
Insufficient physical activity	19.5 (44 / 225)	14.8–25.2	19.9	0.64 (0.45–0.90)	*P* < 0.05
Physically inactive	28.7 (821/ 2856)	27.3–30.7	28.1	1.63 (1.32–2.02)	*P* < 0.0001
Family history of obesity					
Yes	43.0 (376 / 875)	39.7–46.3	41.9	2.70 (2.29–3.19)	*P* < 0.0001
No	21.8 (574 / 2632)	20.1–23.3	21.5	0.37 (0.31–0.44)	*P* < 0.0001
Do not know / No response	22.2 (20 / 90)	13.4–30.6	20.9	NA	NA

^a^Age-adjusted prevalence rates were calculated in relation to the total population of the provinces of Panama and Colon in 2014

^b^For each category of the variables studied, OR and *p* value were calculated between total obese people, and without obesity with relation to the same variable

NA: Not applicable

Source: Prepared by the authors. PREFREC 2010–2011

### Obesity in women

In women, the prevalence of obesity was higher than that estimated for the whole sample (30.9 %, CI 29.2–32.8). The risk of obesity (OR > 1.0) was associated with the following variables: living in urban areas, being 40–59 years old, being Afro-Panamanian, consumption of beverages/foods rich in sugar, being physically inactive, and having a family history of obesity. Almost the totality of obese women (97.9 %), had waist circumference values > 88 cm [OR = 47.5 (28.1–81.6)]; *P* < 0.0001 - Table 2.

**Table 2:** Prevalence of obesity and associated factors in women and men

Variables	Women			Men		
	Prevalence of obesity (%)	O.R.^a^	P value	Prevalence of obesity (%)	O.R.^a^	*P* value
TOTAL	30.9 (757 / 2450)	NA	NA	18.3 (193 / 1057)	NA	NA
Area						
Urban	34.5 (410 / 1188)	1.39 (1.17–1.65)	*P* < 0.0001	23.8 (111 / 467)	1.93 (1.41–2.65)	*P* < 0.0001
Rural	28.0 (322 / 1148)	0.78 (0.65–0.92)	*P* < 0.05	15.2 (77 / 507)	0.67 (0.49–0.92)	*P* < 0.05
Indigenous	21.9 (25 / 114)	0.62 (0.38–0.99)	*P* = 0.04	6.0 (5 / 83)	0.27 (0.09–0.70)	*P* = 0.004
Age (years)						
18–29	19.5 (101 / 519)	0.47 (0.37–0.60)	*P* < 0.0001	12.6 (23 / 182)	0.60 (0.36–0.98)	*P* < 0.05
30–39	33.0 (167 / 506)	1.13 (0.91–1.39)	*P* = 0.25	23.7 (40 / 169)	1.49 (0.98–2.25)	*P* = 0.05
40–49	38.3 (197/ 514)	1.53 (1.24–1.88)	*P* < 0.0001	23.2 (45 / 194)	1.46 (0.98–2.16)	*P* = 0.05
50–59	34.9 (156 / 447)	1.25 (1.00–1.56)	*P* < 0.05	21.5 (44 / 205)	1.29 (0.89–1.96)	*P* = 0.19
60 and over	29.3 (136 / 464)	0.91 (0.73–1.15)	*P* = 0.41	13.3 (41 / 307)	0.61 (0.41–0.90)	*P* < 0.05
Ethnic groups						
Afro-Panamanian	41.9 (228 / 544)	1.88 (1.53–2.30)	*P* < 0.0001	22.5 (45 / 200)	1.39 (0.94–2.06)	*P* = 0.08
Mestizo	29.6 (389 / 1312)	0.88 (0.74–1.05)	*P* = 0.15	19.9 (116 / 582)	1.29 (0.93–1.79)	*P* = 0.12
Native	18.0 (47 / 261)	0.46 (0.32–0.64)	*P* < 0.0001	6.0 (7 / 117)	0.26 (0.11–0.58)	*P* < 0.001
White	29.5 (85 / 288)	0.93 (0.70–1.23)	*P* = 0.59	15.4 (20 / 130)	0.79 (0.46–1.34)	*P* = 0.36
Asian/Others/Not specified	17.8 (8 / 45)	0.48 (0.20–1.07)	*P* = 0.08	17.9 (5 / 28)	0.97 (0.32–2.74)	*P* = 0.84
Intake of fatty foods						
2 or more fried food daily	18.4 (695 / 2219)	1.22 (0.89–1.38)	*P* = 0.19	18.4 (186 / 1008)	1.36 (0.57–3.36)	*P* = 0.46
Less of 2 fried food daily	27.1 (63 / 232)	0.82 (0.60–1.11)	*P* = 0.19	14.3 (7 / 49)	0.74 (0.33–1.66)	*P* = 0.46
Intake of beverage / foods rich in sugars						
6 teaspoons per day or more	33.4 (316 / 946)	1.21 (1.01–1.45)	*P* < 0.05	21.7 (80 / 368)	1.42 (1.02–1.97)	*P* < 0.05
Less of 6 teaspoons per day	29.3 (441 / 1504)	0.83 (0.69–0.98)	*P* < 0.05	19.6 (113 / 576)	0.71 (0.51–0.97)	*P* < 0.05
Physical Activity						
Physically active	25.9 (42 / 167)	0.74 (0.51–1.07)	*P* = 0.09	16.6 (43 / 259)	0.86 (0.58–1.27)	*P* = 0.43
Insufficient physical activity	26.0 (33 / 127)	0.78 (0.51–1.18)	*P* = 0.22	11.2 (11 / 98)	0.54 (0.27–1.07)	*P* = 0.06
Physically inactive	31.6 (682 / 2156)	1.35 (1.01–1.80)	*P* < 0.05	19.6 (139 / 700)	1.39 (0.97–1.99)	*P* = 0.06
Family history of obesity						
Yes	46.4 (308 / 663)	2.59 (2.14–3.13)	*P* < 0.0001	32.1 (68 / 212)	2.72 (1.90–3.90)	*P* < 0.0001
No	25.1 (449 / 1787)	0.39 (0.32–0.47)	*P* < 0.0001	14.8 (125 / 845)	0.38 (0.26–0.51)	*P* < 0.0001
Do not know / No response	22.6 (14 / 62)	NA	NA	22.2 (6 / 27)	NA	NA
Waist Circumference						
>102 cm (Men)	NA	NA	NA	80.0 (155 / 193)	88.8 (53.5–148.4)	*P* < 0.0001
≤102 cm				4.3 (39 / 864)	0.01 (0.01–0.02)	*P* < 0.0001
>94 cm	NA	NA	NA	98.4 (192 / 193)	177.7 (54.4–702.2)	*P* < 0.0001
≤94 cm				26.2 (227 / 864)	0.01 (0.0–0.02)	*P* < 0.0001
>88 cm (Women)	97.9 (741 / 757)	47.5 (28.1–81.6)	*P* < 0.0001	NA	NA	NA
≤88 cm	49.3 (835 / 1692)	0.02 (0.01–0.03)	*P* < 0.0001			

^a^For each category of the variables studied, OR and p value were calculated between total of women/men obese and without obesity with relation to the same variable

The analysis was performed independently for women and men

NA: Not applicable

Source: Prepared by the authors. PREFREC 2010–2011

The logistic regression revealed relationships between women with obesity and family history of obesity (*P* < 0.0001), age (*P* < 0.0001), waist circumference > 88 cm (*P* < 0.0001), ethnic groups (*P* < 0.0001), and area of residence (*P* < 0.0001); log likelihood = 1097.0

### Obesity in men

The prevalence of obesity among men was 18.3 % (CI 15.7–20.3). Obesity was associated (OR > 1.0) with men living in urban areas (*P* < 0.0001), those consuming beverages/foods rich in sugar (*P* < 0.05), and in those with family history of obesity (*P* < 0.0001). 80 % of men with obesity, had waist circumference values > 102 cm (central obesity) [OR = 88.8 (53.5–148.4)]; *P* < 0.0001; however, when we used the waist circumference cut–off point > 94 cm the prevalence of obesity increased to 98.4 % [OR = 177.7 (54.4–702.2)]; *P* < 0.0001 - Table 2.

The logistic regression revealed relations between obesity in men and area of residence (*P* < 0.0001), family history of obesity (*P* < 0.001), waist circumference > 102 cm (*P* < 0.0001), and ethnic groups (*P* < 0.05); log likelihood = 532.4.

### Diseases associated to obesity

Among people with obesity, 12.0 % suffered from T2DM, 43.8 %, from hypertension and 73.5 % from LDL values ≥ 100 mg/dL. In contrast, 39.4 % of individuals with T2DM were obese, same as 34.6 % of hypertensive subjects; 33.0 % of women with HDL values < 40 mg/dL, 22.3 % of men with HDL values < 50 mg/dL; and, 30.0 % of the individuals with LDL values ≥ 100 mg/dL.

For women and men, obesity was a risk factor associated to T2DM, hypertension, LDL values ≥ 100 mg/dL, and low HDL values (<50 mg/dL for women and < 40 mg/dL for men), OR > 1.0; *P* < 0.05 – Table 3.

**Table 3:** Prevalence of diseases associated with obesity in women and men

Variables	Total of People with Obesity				Women			Men		
	Prevalence (%)	Confidence Intervals (95 %)	O.R.^a^	P value	Prevalence of obesity (%)	O.R.^a^	P value	Prevalence of obesity (%)	O.R.^a^	P value
Diabetes Mellitus										
Yes	12.0 (114 / 950)	9.9–14.1	1.86 (1.45–2.38)	*P* < 0.001	10.7 (81 / 757)	1.66 (1.23–2.24)	*P* < 0.001	17.1 (33 / 193)	2.71 (1.72–4.28)	*P* < 0.001
No	88.0 (836 / 950)	85.6–89.9								
High blood pressure										
Yes	43.8 (416 / 950)	40.8–47.2	1.75 (1.50–2.04)	*P* < 0.001	41.7 (316 / 757)	1.91 (1.60–2.29)	*P* < 0.001	51.8 (100 / 193)	1.77 (1.30–2.43)	*P* < 0.001
No	56.2 (534 / 950)	53.1–59.5								
LDL										
≥100 mg/dL	73.5 (698 / 950)	70.2–75.8	1.58 (1.34–1.87)	*P* < 0.001	72.9 (552 / 757)	1.58 (1.31–1.91)	*P* < 0.001	75.6 (146 / 193)	1.66 (1.17–2.40)	*P* < 0.05
<100 mg/dL	26.5 (252/ 950)	23.3–29.0								
HDL < 50 mg/dL (Women)	NA	NA	NA	NA	89.7 (679 / 757)	1.99 (1.53–2.59)	*P* < 0.001	NA	NA	NA
HDL < 40 mg/dL (Men)	NA	NA	NA	NA	NA	NA	NA	83.9 (163 / 193)	2.89 (1.91–4.37)	*P* < 0.001

^a^For each category of the variables studied, OR and p value were calculated between total of women/men obese and without obesity with relation to the same variable

NA: Not applicable

Source: Prepared by the authors. PREFREC 2010–2011

## Discussion

This is the first epidemiological study conducted in Panama in which obesity and its risk factors were studied in representative samples. A strict methodology was employed to reduce bias and to ensure that the data were precise and accurate [14].

The prevalence of obesity found in this research is higher than that reported for the country (20.4 %) by the National Survey of Living Standards 2 008 [20]; it was compared with that of other countries in the region and was found to be higher than that of Nicaragua, 2 009 (22.0 %) [21]; Peru, 2 006 (11.4 %) [22]; Argentina, 2 005 (14.6 %) [23] and Uruguay, 2 004 (17.7 %) [24] but was lower than that estimated in the United States of America (U.S.A.) by the NHANES study in 2 011–2 012 (34.9 %) [25] as well as that of Mexico by the National Health Survey 2 012 (32.4 %) [26].

As reported in other studies [3, 20–22, 24–32], the prevalence of obesity in women was higher than that of men, independently of the age. Some studies attribute this fact to female physiological hormonal cycle [33]; others to pregnancy, where evidence suggests that more than 70 % of women held 10 Kg or more of weight after delivery, independently of age and race [34].

According to the age groups studied, an increase in risk of obesity occurs after age 29, being higher in women than in men. In men aged 60 years, the prevalence decreased to values similar to those of the 18–29-year-old group; in contrast, in women aged 60 and more an elevated prevalence of obesity was still present, increasing the risk for the development of other chronic diseases (i.e. T2DM, hypertension, stroke), as well as mortality.

Our study evidenced that being Afro-Panamanian (African-American) was a risk factor for obesity only in women. Similarly reported by other authors [26, 32, 35], an increased prevalence of obesity for both sexes was found in this ethnic group, where the high consumption of fatty meals is deeply rooted in the Afro-Caribbean culture and contributes to the development of obesity [14].

Significant nutritional and lifestyle changes have happened in the Panamanian society in the last 3 decades [27, 36–39]; the per capita gross domestic product of Panama increased almost five fold and the percentage of people living in urban areas grew from 50 to 75 % [39–41]. This rapid increase in economic growth and urbanization has lead to changes in occupation, transportation and technology directed at leisure time activities at home which have also contributed to increased sedentary behavior and reduced physical activity [39, 42, 43].

In U.S.A., as reported in the NHANES 2 005–2 008 study [30], the highest proportion of individuals with obesity was located in rural areas, while in Panama, the highest proportion of participants with obesity was in urban areas, although the prevalence of obesity in both areas of the U.S.A. are higher than those observed in Panama. In Panamanian indigenous areas, there was a lower prevalence of obesity, which can be attributed to increased physical activity, less sedentary lifestyles and more natural food consumption.

Several studies found a relationship between the intake of saturated and trans fats [44–46] as well as the intake of a diet with high caloric density [45] and weight gain. In our study, the intake of fatty foods was very high, and these characteristics were closely linked to obesity and cardiovascular problems. Although these results were not statistically significant in men and women, frying foods is a common habit in the Panamanian population, reason why it must be considered in the multicausal analysis of obesity.

Our study found a statistically significant association with the daily consumption of beverages/foods with an elevated concentration of sugar and obesity, for both sexes. These results are similar to those reported by the Nurses’ Health Study II [47]; a study of UK adults [48] and a meta-analysis published in the American Journal of Public Health [49]. Similar results were found in the Framingham Heart Study, where the consumption of one or more sugary drinks per day was associated with an increased risk of obesity and increased waist circumference [50].

The food consumption behavior mentioned above, can be explained by the fact that almost 30 % of the Panamanian population is considered “poor” (poor is being defined as a monthly income of less than 98.00$ per family) [51]. In this regard, the food industry furnishes several types of “socially accepted” meals, which have a high energetic density (rich in sugars and fat), a good flavor, a great satiety effect, and a very low cost [52]. Additionally, this people have limited access to fruits, vegetables and foods with a high amount of fiber (foods of a good nutritional quality).

Similarly, as reported in the literature [53], the highest prevalence of obesity was among those who performed less than 60 min of physical activity per week, being a risk factor in men and women. We also observed a higher prevalence of this disease among individuals with a family history of obesity, which may reflect the influence of genetics in the development of this disease [1, 4, 12, 35, 52, 53].

As seen in our results, 80 % and more of the obese population had a waist circumference above the WHO cut-off points for abdominal obesity; although being the most used cut-off points, they’re targeted for Caucasian populations and not for Hispanic populations like ours. This fact encouraged us to adjust the male waist circumference cut-off point for Hispanics (>94 cm), obtaining the, not-surprising result, that almost all of the male obese participants (98.3 %) were over this cut-off point. In this regard, we strongly believe that the information gathered, could be used to assist in the development of more specific cut-off points for the diagnosis of obesity in Panamanian populations [54].

In this regard, several studies suggest that even a slight abdominal accumulation of adipose tissue is associated with higher rates of cardiovascular disease and other conditions [1–4, 6, 8, 10–12] than with overweight only and obesity only [54–56]. In Panama, the elevated prevalence of central obesity represents a very significant risk factor in developing and increasing comorbidity as of T2DM, hypertension, reduction in HDL values and LDL values ≥ 100 mg/dL, particularly in men. Obesity measured by BMI, explains only one-third of the total variation in insulin sensitivity, which is more strongly correlated with central obesity and with the development of T2DM [8, 55].

Additionally, if BMI raises above 30 kg/m^2^, HDL levels decline in a linear fashion, more strongly and significantly [57]. Similarly, as BMI increases, systolic and diastolic blood pressure rises; although the causes are unknown, this is probably due to elevated insulin concentrations (resulting from insulin resistance), which are conducive to renal sodium retention, higher plasma renin concentrations and an increase in catecholamine activity [8, 55].

### Limitations

This study has several limitations. PREFREC is a cross-sectional study; hence food consumption and physical activity measures do not necessarily represent usual patterns over time that led to the individual’s nutritional status at the time of the survey.

This study might have errors of measurement and parallax for height, weight and waist circumference. However, the elaboration of this research involved the development of three workshops for the standardization of anthropometric measures attended by our team of nutritionists, which were the only ones who performed all of the measurements.

The ratio of female–male responses was 2.3:1, with an even higher ratio for those under 40 years of age. This result reflects selection bias, which may be related to the type of sampling strategy used (stratified according to education level); the cultural traditions in the country and the greater acceptance of women participating in population-based research; or the requirement to abstain from alcohol for 24 h before the survey, which may have lowered the participation rate among men.

Due to the cross-sectional nature of this study, the self-reported variables (food consumption and performance of physical activity), may inquire into bias, as it is difficult for the participants to remember with precision the foods consumed in a prior week, as well as physical activity. On the other hand, it is important to emphasize that obesity, as well as many other chronic diseases, are not transversal processes, in this regard, the report of a particular habit in a particular time, may not reflect the consumption behavior of the person when the pathologic process was in its development phase.

PREFREC, is not a national study, however, it represents 60.4 % of Panama’s total population of 18 years and over.

## Conclusions

Obesity represents a serious public health concern with a sex disparity in the Panamanian population, affecting more women than men.

Living in an urban area, being 30 years of age or older, being Afro-Panamanian, engaging in less than 60 min of physical activity per week, having a family history of obesity and consuming six or more teaspoons of sugar in beverages and/or foods rich in sugars daily, represented risk factors associated with obesity in women. The same risk factors were associated with obesity in men, with the exception of the Afro-Panamanian ethnic group.

Previously conducted studies in women found a relationship between the intake of saturated and trans fats [44–46, 48] as well as the intake of diets with high caloric density [45] and weight gain. In PREFREC, intake of fatty foods was considered very high, and these characteristics were closely linked to obesity and cardiovascular problems. Although these results were not statistically an elevated consumption of fats, fried foods and snacks, could substantially contribute to the prevalence of obesity in the Panamanian population.

Our study confirms a direct association in Panama between excess weight, hypertension, T2DM, LDL values ≥ 100 mg/dL and low HDL values for women and men (<50 mg/dL and < 40 mg/dL, respectively), underscoring the heightened risk for these risk factors in people with obesity compared to individuals without obesity.

This information is quite significant, as it will aid strengthening primary prevention programs (public health policies) targeting obesity in specific populations groups, especially those groups of individuals with a family history of obesity and of Afro-Panamanian ethnic groups; additionally, tertiary prevention programs should be implemented for obese women who are 40–59 years of age living in urban areas and who have a family history of obesity [58].

Although this investigation was conducted between October 2010 to January 2011 and due to the absence of previous and posterior cardiovascular surveys, our study can be used as a country indicator in order to produce a more accurate record of the prevalence of obesity in Panama among adults and to measure its impact as a risk factor when present with other co-morbidities.

## Abbreviations

WHO: World Health Organization
BMI: Body Mass Index
GMI: Gorgas Memorial Institute for Health Research
PREFREC for its acronym in Spanish: Survey on Risk Factors Associated with Cardiovascular Disease
INEC: The National Institute of Statistics and Census
CI: Confidence intervals
SD: Standard deviation
OR: Odds Ratio
NHANES: National Health Survey of the United States
T2DM: Type 2 Diabetes Mellitus
LDL: Low Density Lipoprotein Cholesterol
HDL: High Density Lipoprotein Cholesterol
FPHC: Female Physiological Hormonal Cycle
